# A 28-Year History of HIV-1 Drug Resistance and Transmission in Washington, DC

**DOI:** 10.3389/fmicb.2019.00369

**Published:** 2019-03-08

**Authors:** Keylie M. Gibson, Margaret C. Steiner, Seble Kassaye, Frank Maldarelli, Zehava Grossman, Marcos Pérez-Losada, Keith A. Crandall

**Affiliations:** ^1^Computational Biology Institute, Milken Institute School of Public Health, George Washington University, Washington, DC, United States; ^2^Department of Medicine, Georgetown University, Washington, DC, United States; ^3^HIV Dynamics and Replication Program, Host-Virus Interaction Branch, Center for Cancer Research, National Cancer Institute, National Institutes of Health, Bethesda, MD, United States; ^4^Sackler Faculty of Medicine, School of Public Health, Tel Aviv University, Tel Aviv, Israel; ^5^CIBIO-InBIO, Centro de Investigação em Biodiversidade e Recursos Genéticos, Universidade do Porto, Vairão, Portugal; ^6^Department of Epidemiology and Biostatistics, Milken Institute School of Public Health, George Washington University, Washington, DC, United States

**Keywords:** Washington, DC, phylodynamics, HIV-1, drug resistance mutations, transmission networks

## Abstract

Washington, DC consistently has one of the highest annual rates of new HIV-1 diagnoses in the United States over the last 10 years. To guide intervention and prevention strategies to combat DC HIV infection, it is helpful to understand HIV transmission dynamics in a historical context. Toward this aim, we conducted a retrospective study (years 1987–2015) of 3,349 HIV *pol* sequences (1,026 bp) from 1,995 individuals living in the DC area belonging to three different cohorts. We coupled HIV sequence data with clinical information (sex, risk factor, race/ethnicity, viral load, subtype, anti-retroviral regimen) to identify circulating drug resistant mutations (DRM) and transmission clusters and assess their persistence over time. Of the transmission clusters identified in the DC area, 78.0 and 31.7% involved MSM and heterosexuals, respectively. The longest spread of time for a single cluster was 5 years (2007–2012) using a distance-based network inference approach and 27 years (1987–2014) using a maximum likelihood phylogenetic approach. We found eight subtypes and nine recombinants. Genetic diversity increased steadily over time with a slight peak in 2009 and remained constant thereafter until 2015. Nucleotide diversity also increased over time while relative genetic diversity (BEAST) remained relatively steady over the last 28 years with slight increases since 2000 in subtypes B and C. Sequences from individuals on drug therapy contained the highest total number of DRMs (1,104–1,600) and unique DRMs (63–97) and the highest proportion (>20%) of resistant individuals. Heterosexuals (43.94%), MSM (40.13%), and unknown (44.26%) risk factors showed similar prevalence of DRMs, while injection drug users had a lower prevalence (33.33%). Finally, there was a 60% spike in the number of codons with DRMs between 2007 and 2010. Past patterns of HIV transmission and DRM accumulation over time described here will help to predict future efficacy of ART drugs based on DRMs persisting over time and identify risk groups of interest for prevention and intervention efforts within the DC population. Our results show how longitudinal data can help to understand the temporal dynamics of HIV-1 at the local level.

## Introduction

The prevention and treatment of HIV in Washington, DC is a public health priority both locally and nationally, as more diagnoses per unit population occur annually in the District than in any of the 50 states (Centers for Disease Control and Prevention, 2017). According to the most recent report by the DC Department of Health (DOH), over 1,900 per 100,000 residents are living with HIV in DC as of 2017 (District of Columbia Department of Health, HIV/AIDS, Hepatitis, STD and TB Administration (HAHSTA), 2018). This surpasses the WHO's threshold for a generalized epidemic (1%) (District of Columbia Department of Health, HIV/AIDS, Hepatitis, STD and TB Administration (HAHSTA), 2017). Additionally, the epidemic disproportionately affects black men and women, who comprise 75% of HIV cases but only 46.7% of residents in DC (District of Columbia Department of Health, HIV/AIDS, Hepatitis, STD and TB Administration (HAHSTA), 2017). Seven of the eight wards in DC have an HIV prevalence greater than 1%, indicating that the epidemic is not entirely geographically localized (Pérez-Losada et al., 2017). Similar to the Chicago HIV epidemic (Morgan et al., 2017), it has been proposed that the severity of the DC epidemic may be due to the high proportion of residents who belong to high risk groups and the city's considerable economic stratification (Greenberg et al., 2009).

The United States HIV epidemic is composed of predominantly subtype B variants (Vermund and Leigh-Brown, 2012), although several studies report that non-B subtypes are becoming more prominent over time both in DC (Grossman et al., 2018), the US (Germer et al., 2015) and globally (Ivanov et al., 2013; Neogi et al., 2014; UK Collaborative Group on HIV Drug Resistance, 2014; Esbjörnsson et al., 2016). Notably, of the individuals living with HIV in DC, more than 10% were born outside of the United States, thus leading to a potentially high diversity of HIV in the region (Grossman et al., 2018). Additionally, with a variety of subtypes and recombinant forms circulating in Washington, DC, the presence of drug resistant mutations (DRM) becomes a relevant clinical issue that needs to be addressed in order to provide efficient and effective treatment. Drug resistance is one of the most pressing issues in HIV treatment today, as resistance to preventative and treatment drugs affects the ability of the virus to evade the immune system and persist in the infected individual. Drug resistant mutations may occur at the time of transmission, so treatment-naïve individuals may also be resistant at the time of diagnosis. Currently, there are 97 known codons affected by DRMs in the Stanford HIV database (https://hivdb.stanford.edu), all either conferring resistance to or under surveillance (SDRM) for one or more of the current 22 antiretroviral (ART) drugs. Resistance to antiretroviral drugs and ensuing viremia can result in immunosuppression leading to morbidity, and, if new DRMs arise, then new drugs are needed to combat the ever-evolving virus. In 2016, Kassaye et al. found a high prevalence of drug resistance mutations in persons living with HIV in DC. Thus, studying the presence and spread of DRMs in Washington, DC can provide a temporal and spatial context to study the dynamics of HIV drug resistance. Furthermore, identifying DRMs present and spreading between risk groups will provide public health officials with information to implement more efficient targeted education and prevention efforts and programs.

According to the DC DOH, men who have sex with men (MSM) and heterosexuals (HRH) are the primary modes of HIV transmission in DC, while transmission via injection drug use (IDU) has decreased by 95% from 2007 to 2017 (District of Columbia Department of Health, HIV/AIDS, Hepatitis, STD and TB Administration (HAHSTA), 2017). About 27% of people living with HIV are black MSM, while the second and third most prominent HIV risk groups are heterosexual black women and white MSM (District of Columbia Department of Health, HIV/AIDS, Hepatitis, STD and TB Administration (HAHSTA), 2017). A cross-sectional study in 2009 identified heterosexual transmission as an emerging epidemic in non-Hispanic black men and women in DC, demonstrating that this risk is based on transmission networks rather than individual risk (Magnus et al., 2009). Similarly, a study of transmission clusters in Sweden, Denmark, and Finland suggested MSM-heterosexual transmission played a significant role in HIV infection since the majority of the mixed-transmission clusters involved individuals from those two risk groups (Esbjörnsson et al., 2016).

To address the current HIV epidemic in Washington, DC—and to better recognize how it will evolve in the future—we must first understand the evolutionary dynamics of the local epidemic. Toward this aim, we conducted a retrospective study (years 1987–2015) of 1,995 HIV-infected individuals whose 3,349 sequences were collected from three separate datasets. Our analysis focused specifically on the polymerase (*pol)* gene, the most frequently sequenced gene in HIV-1 clinical studies of DRMs. We identified transmission clusters with associations to demographic information and mapped epidemiological variables on estimated transmission clusters. Lastly, we identified drug resistance mutations and assessed their variation over time, as well as estimated sequence diversity over time.

## Materials and Methods

### Datasets

A total of 3,349 HIV sequences (after removing duplicates) from the metropolitan DC area (including Washington, DC, northern Virginia, and northern and southern Maryland) were combined from three independently collected datasets: Pérez-Losada et al. (2017) with 1,659 sequences one each from that same number of individuals, Maldarelli et al. (2013) with 1,387 sequences collected from 33 individuals, and new HIV data presented here representing 303 sequences, one per individual (Table 1). All duplicate sequences in Pérez-Losada et al.'s dataset were removed, but all sequences in Maldarelli et al.'s dataset were used as it included known intra-patient variation and reflected the evolution of the virus over time. All studies performed DNA Sanger sequencing. Collectively, we have a total of 1,995 patients with sequence samples obtained between 1987 and 2015. Demographic variables considered were: sex, gender, race/ethnicity, age, country of birth, state of residence, risk factor, viral load, duration of infection, CD4+T lymphocyte count, HIV-1 subtype, and antiretroviral regimen type. These characteristics were paired with their respective HIV-1 sequence(s) (Table 1).

**Table 1:** Phenotypic characteristics of each dataset separated and combined.

	**(Maldarelli et al., 2013)**	**(Pérez-Losada et al., 2017)**	**This study**	**Total**
Number of individuals	33	1,659	303	1,995
Number of sequences	1,387	1,659	303	3,349
Median age (years)	35.5	46	39	44.79
Age range (years)	19.5, 51.5	10, 82	20.2, 75	10, 82
Quartiles (years)	28.5, 41.9	34, 54	31.8, 46.5	30, 50
**RACE/ETHNICITY**				
Black	NA	1,394 (84.0%)	NA	1,394 (69.9%)
White	NA	138 (8.3%)	NA	138 (6.9%)
Hispanic	NA	76 (4.6%)	NA	76 (3.8%)
Other	NA	22 (1.3%)	NA	22 (1.1%)
Unknown	33 (100%)	29 (1.8%)	303 (100%)	365 (18.3%)
**SEX AT BIRTH**				
Male	NA	1,117 (67.3%)	NA	1,117 (56.0%)
Female	NA	542 (32.7%)	NA	542 (27.2%)
Unknown	33 (100%)	0 (0%)	303 (100%)	336 (16.8%)
**GENDER**				
Male	31 (93.9%)	1,117 (67.3%)	229 (75.6%)	1,377 (69.0%)
Female	2 (6.1%)	543 (32.7%)	72 (23.8%)	617 (30.9%)
Transgender	0 (0%)	0 (0%)	2 (0.7%)	2 (0.1%)
Unknown	0 (0%)	0 (0%)	0 (0%)	0 (0%)
**COUNTRY OF BIRTH**				
US	NA	114 (6.9%)	NA	114 (5.7%)
Non-US	NA	50 (3.01%)	NA	50 (2.5%)
Unknown	33 (100%)	1,495 (90.1%)	303 (100%)	1,831 (91.8%)
**STATE OF RESIDENCE**				
DC	NA	1,368 (82.5%)	303 (100%)	1,671 (83.8%)
MD	NA	127 (7.7%)	0 (0%)	127 (6.4%)
VA	NA	28 (1.7%)	0 (0%)	28 (1.4%)
Other	NA	0 (0%)	0 (0%)	0 (0%)
Unknown	33 (100%)	136 (8.2%)	0 (0%)	169 (8.5%)
**HIV RISK FACTOR**				
MSM	0 (0%)	637 (38.4%)	138 (45.5%)	775 (38.8%)
IDU	0 (0%)	86 (5.2%)	6 (1.98%)	92 (4.61%)
Heterosexual	0 (0%)	558 (33.6%)	152 (50.2%)	710 (35.6%)
Sex	33 (100%)	0 (0%)	2 (0.7%)	35 (1.75%)
Other	0 (0%)	378 (22.8%)	3 (1.0%)	381 (19.1%)
Unknown	0 (0%)	303 (18.3%)	2 (0.7%)	305 (15.3%)
Median CD4 count (cells/μl)	401	498	274.5	419
**VIRAL LOAD (COPIES/ML)**				
< 200	NA	129 (7.8%)	39 (12.9%)	168 (%)
200–399	NA	32 (1.9%)	4 (1.3%)	36 (%)
400–9,999	NA	245 (14.8%)	63 (20.8%)	308 (%%)
>10,000	NA	371 (22.4%)	154 (50.8%)	525 (%)
Unknown	33 (100%)	882 (53.2%)	43 (14.2%)	958 (48.0%)
**ART EXPOSURE**				
Experienced	0 (0%)	1,005 (60.6%)	0 (0%)	1,005 (50.4%)
Naïve	0 (0%)	106 (6.4%)	303 (100%)	409 (20.4%)
Unknown	33 (100%)	548 (33.0%)	0 (0%)	581 (29.1%)
**ART REGIMEN TYPE**				
Single Class				
NRTI	NA	5 (0.3%)	NA	5 (0.3%)
NNRTI	NA	2 (0.1%)	NA	2 (0.1%)
PI	NA	6 (0.4%)	NA	6 (0.3%)
ENH	NA	4 (0.2%)	NA	4 (0.2%)
INT	NA	7 (0.4%)	NA	7 (0.4%)
Dual Class	NA	59 (3.6%)	NA	59 (3.0%)
Multiple Class	NA	1,455 (87.7%)	NA	1,455 (72.9%)
Unknown	33 (100%)	121 (7.3%)	NA	154 (7.7%)
DRM Present	438 (31.6%)	647 (39.0%)	185 (61.1%)	1,270 (37.9%)
**SUBTYPE**				
B	33 (100%)	1,559 (93.9%)	225 (74.3%)	1,817 (91.1%)
C	0 (0%)	32 (1.9%)	16 (5.3%)	48 (2.4%)
D	0 (0%)	7 (0.4%)	4 (1.3%)	11 (0.6%)
B recombinant	0 (0%)	38 (2.3%)	21 (6.9%)	59 (2.9%)
Other	0 (0%)	23 (1.4%)	37 (12.2%)	60 (3.0%)
**SEQUENCING DATE**				
1987–1994	1 (3.0%)	0 (0%)	0 (0%)	1 (0.05%)
1995–2000	14 (42.4%)	0 (0%)	7 (2.3%)	21 (1.1%)
2001–2005	18 (54.5%)	0 (0%)	45 (14.9%)	63 (3.2%)
2006–2010	0 (0%)	0 (0%)	112 (37.0%)	112 (5.6%)
2011–2015	0 (0%)	1,659 (100%)	139 (45.9%)	1,798 (90.1%)

*DC, District of Columbia; MD, Maryland; VA, Virginia; MSM, men who have sex with men; IDU, injection drug users; ART, antiretroviral therapy; DRM, drug resistance mutations; NRTI, nucleoside reverse transcriptase inhibitors; NNRTI, non-nucleoside reverse transcriptase inhibitors; PI, protease inhibitors; ENH, enhancers; INT, integrase inhibitors*.

Briefly, sequences in the Pérez-Losada et al. (2017) dataset were collected through the DC Cohort, a longitudinal study conducted by the DC Department of Health at 13 DC area clinics (Milken Institute School of Public Health, 2018). Over 1,700 patients were enrolled in this study between 2011 and 2015. RNA from plasma samples was sequenced by LabCorp as part of routine clinical care with a focus on the *pol* genes. RT-PCR and Sanger sequencing were used to generate reads, which were analyzed with Sequencher DNA Sequence Analysis Software. Sequences for *PR/RT* sequences had a length of 1,496 bp. This dataset included additional integrase sequences (864 bp), which were not included in our analysis.

The dataset originally published in Maldarelli et al. (2013) was comprised of 33 patients treated at the NIH Clinical Center in Bethesda, Maryland. HIV sequences were obtained via single genome Sanger sequencing from plasma samples; limiting dilution was completed on each plasma sample. These patients were sampled longitudinally. Amplicons covering protease and reverse transcriptase (297 and 700–1200 bp, respectively) were used to obtain *PR/RT* sequences.

Data collection and sequencing of new HIV data was completed with the same single genome sequencing procedure as in Maldarelli et al. (2013). However, longitudinal sampling of these patients was not completed. A single plasma sample was taken from each patient at time of sampling and only a single limiting dilution was completed. These patients were also from the NIH Clinical Center in Bethesda, MD.

### Phylodynamic Analyses

We aligned all sequences to the HXB2 reference *pol* sequence using MAFFT (Katoh et al., 2002). Aligned sequences were trimmed to a 1,026 bp region (2,253–3,279 bp relative to HXB2; GenBank accession K03455), which covered all of *PR* and a region of *RT* (55%). Any nucleotides outside this gene region were trimmed to ensure consistency among datasets. We used jModelTest (Posada, 2008) to estimate the best-fit model of molecular evolution along with model parameter values used in phylogenetic inference (Posada and Crandall, 2001). We constructed a maximum-likelihood (ML) phylogenetic tree using RAxML v. 8.2.9 (Stamatakis, 2014).

We assessed transmission clusters using both phylogenetic analyses and the network method HIV-TRACE (Kosakovsky Pond et al., 2018). HIV-TRACE identifies transmission clusters by analyzing genetic distance between pairs of sequences. For HIV-TRACE, we used a genetic distance threshold of 0.01 substitutions/site (Wertheim et al., 2017; as in Pérez-Losada et al., 2017) to account for the genetic diversity of *pol*. Additionally, we handled the ambiguities in the sequences using an average to account for all possible resolutions and avoid biases and false positive transmission clusters between the different datasets and sequencing strategies. Default settings were used for the remaining parameters. For the ML phylogenetic method, a transmission cluster was defined as a node with ≥70 bootstrap support (Felsenstein, 1985).

Watterson's genetic diversity (θ), nucleotide diversity (π), haplotype diversity (h), and the number of polymorphic sites (S) were estimated separately for HIV subtypes B and C and recombinant B and patient subsets (e.g., risk types, sex, race/ethnicity, date, and dataset) in DnaSP v. 6.11.01 (Rozas et al., 2017). Relative genetic diversity over time was inferred separately for subtypes B and C and recombinant B in BEAST2 (Bouckaert et al., 2014) using the GMRF Bayesian Skyride model (Minin et al., 2008) and the date of the HIV-1 sample as calibration and a normal prior with mean = 0.001 and *SD* = 0.0005 for ucld.mean. Additionally, we used the HKY substitution model with gamma-distributed among-site rate heterogeneity, a relaxed clock (log-normal) model of rate of substitution and partitioned into three codon positions. We performed two runs 5 × 10^8^ generations each with sampling every 5,000 generations. Parameter uncertainty was summarized in the 95% highest posterior density (HPD) intervals. Results generated by BEAST were visualized in Tracer (Rambaut et al., 2018) with a 10% burn-in.

### Drug Resistance Mutations and Subtype Analyses

We identified drug resistance mutations using the HIVdb program (Liu and Shafer, 2006) from the Stanford University HIV Drug Resistance Database (https://hivdb.stanford.edu). HIVdb identifies known resistance and surveillance variants (SDRMs) associated with 22 approved antiretroviral therapies including Protease Inhibitors (PI), Nucleoside RT Inhibitors (NRTI), Non-Nucleoside RT Inhibitors (NNRTI), and Integrase Inhibitors (INSTI), as well as treatment-selected mutations (TSMs). In order to compare DRMs over time, the number of affected codons for sequences from a single year was normalized by the number of sequences from that year in the dataset. We identified HIV-1 subtypes and recombinants phylogenetically using 170 subtype and recombinant reference sequences from the Los Alamos HIV database (http://www.hiv.lanl.gov/) and ML tree reconstruction, as well as using the REGA subtyping tool v. 3.0 provided by Stanford University (Pineda-Peña et al., 2013). Although subtyping was already reported for the Pérez-Losada et al. dataset, we included these sequences again for confirmation and comparison to the additional datasets from Washington, DC.

## Results

The number of sequences included in our analyses varied between 0 and 540 sequences per year, with seven years missing between 1987 and 2015. The cohort was comprised primarily of men who have sex with men (MSM; 38.8%) and high-risk heterosexuals (HRH; 35.6%) (Table 1). The median age at the time of sequencing was 44.8 years, with lower and upper bounds of 10.0 and 82.0 years old, respectively. Antiretroviral exposure information was only available for Pérez-Losada et al.'s dataset, and the majority of those patients were on multiple-class drug regimens (57.93%). REGA and phylogenetic subtyping methods identified eight unique HIV-1 subtypes and nine recombinants in our combined dataset, of which 10 were neither B or B recombinants (BD, CRF12_BF, DB, CRF24_BG, BF, and BA). Those subtypes were B (94.95%), C (1.43%), and A, D, G, F1, F2, J, (< 1%) and those recombinants were BD (1.37%), and CRF02_AG, CRF12_BF, DB, CRF18_cpx, CRF24_BG, BF, BA, and AK (< 1%).

**Figure 1:**
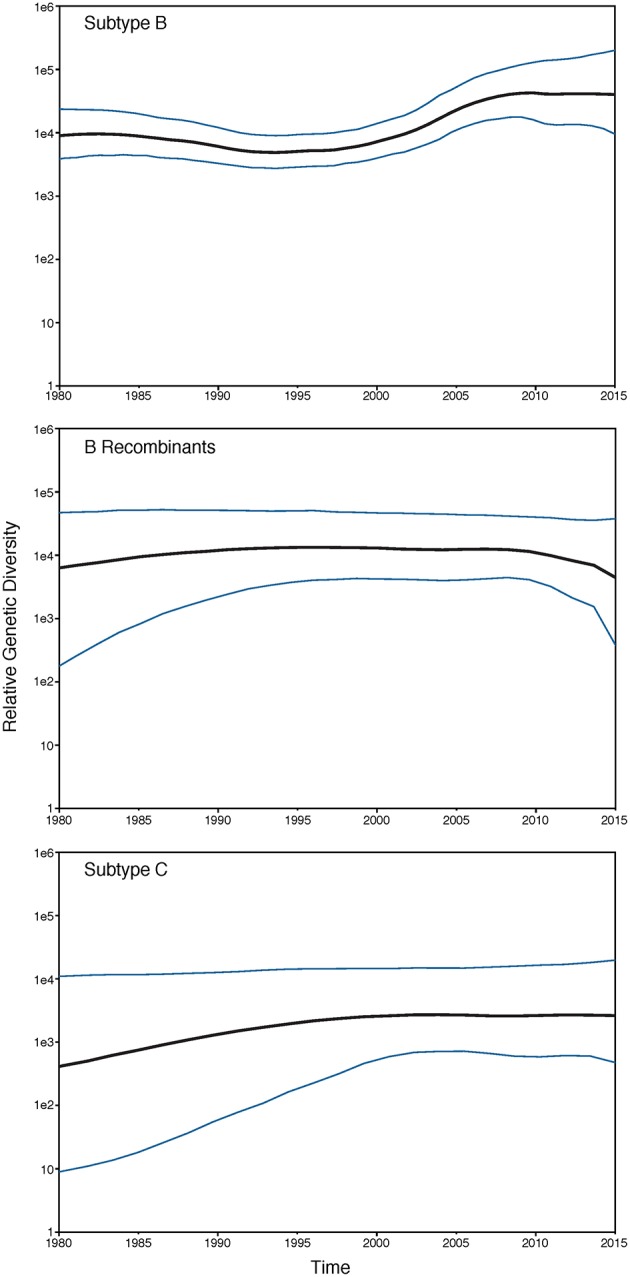
GMRF Bayesian Skyride plot of HIV-1 subtypes past population dynamics. The 95% high posterior density limits of the relative genetic diversity over time are shown in blue. Black lines show the median estimate of the relative genetic diversity over time.

Overall, Washington, DC was found to have a high genetic diversity (mean Watterson's θ = 0.082, Table 2), with B recombinants having a slightly higher genetic diversity (θ = 0.089) compared to subtype B (θ = 0.079) and subtype C (θ = 0.079) (Table 2). For all subtypes, males had a higher genetic diversity than females, and for subtype B, all races/ethnicities had similar genetic diversity. However, for B recombinants and subtype C, blacks had a higher genetic diversity than the remaining races/ethnicities. For subtype B, there was an overall high genetic diversity for all risk groups, with general sexual contact (SEX) risk having the lowest genetic diversity (θ = 0.079). This is not unexpected given that all 1,387 HIV sequences from patients with general sexual contact risk belong to Maldarelli et al.'s dataset and are only from 33 patients. Furthermore, subtype B injection drug users (IDU) had the lowest nucleotide diversity (π = 0.046), while MSM and HRH had similar nucleotide diversity estimates. Moreover, genetic diversity in subtype B increased steadily over time, with a slight decrease in diversity in 2003 and 2004 (Table 2). Nucleotide diversity increased over time with a peak in 2005 and 2006. No steady trends of diversity were estimated in B recombinants and subtype C. Additionally, the past demographic analyses of subtype B, B recombinants, and subtype C in BEAST (Figure 1) indicated that HIV relative genetic diversity remained stable over the last 35 years, with subtype B showing more variability.

**Table 2:** Nucleotide and genetic diversity estimates.

	**Subtype B**					**B Recombinants**					**Subtype C**				
**Subset**	***N***	***h***	***S***	**π**	**θ (W)**	***N***	***h***	***S***	**π**	**θ (W)**	***N***	***h***	***S***	**π**	**θ (W)**
All	3085	50	1005	0.05323	0.07934	156	103	522	0.0656	0.08882	48	44	362	0.05582	0.07875
**DATASETS**															
Frank	1387	1045	851	0.04975	0.07219	0	NA	NA	NA	NA	0	NA	NA	NA	NA
NIH	222	54	497	0.05587	0.08414	34	34	351	0.0647	0.0883	16	16	208	0.0533	0.06561
LC	1476	60	656	0.05612	0.0812	122	114	494	0.06612	0.09006	32	32	314	0.05691	0.07693
**GENDER**															
Male	2503	60	983	0.05215	0.0788	112	105	493	0.06581	0.08914	26	26	287	0.05554	0.07287
Female	580	43	607	0.0506	0.07563	43	43	379	0.0644	0.08674	22	22	258	0.05576	0.06902
Transgender	1	NA	NA	NA	NA	1	NA	NA	NA	NA	0	NA	NA	NA	NA
Unknown	1	NA	NA	NA	NA	0	NA	NA	NA	NA	0	NA	NA	NA	NA
**RACE**															
Black	1244	65	644	0.05569	0.08212	101	96	477	0.06596	0.0902	25	25	277	0.05559	0.07258
Hispanic	64	64	428	0.0577	0.08879	9	9	198	0.06333	0.07169	2	2	63	0.06294	0.06294
White	128	116	483	0.05622	0.08723	7	7	176	0.06499	0.07083	0	NA	NA	NA	NA
Other	17	17	302	0.06634	0.08759	1	NA	NA	NA	NA	3	3	95	0.06348	0.06217
Unknown	1632	140	653	0.04904	0.08001	38	38	363	0.06503	0.08703	18	18	224	0.05731	0.06854
**RISK FACTOR**															
HRH	572	39	596	0.05568	0.08175	60	60	416	0.06327	0.08594	32	31	309	0.05493	0.07448
IDU	89	78	429	0.04635	0.08106	3	3	89	0.06022	0.05957	0	NA	NA	NA	NA
MSM	690	74	616	0.05669	0.08216	76	75	452	0.06571	0.08832	4	4	100	0.05518	0.05421
MSM/IDU	17	17	266	0.05718	0.07743	1	NA	NA	NA	NA	0	NA	NA	NA	NA
SEX	1387	1045	851	0.04975	0.07219	0	NA	NA	NA	NA	0	NA	NA	NA	NA
Other	57	56	435	0.06141	0.09184	3	3	125	0.08447	0.08447	0	NA	NA	NA	NA
Unknown	273	75	529	0.05409	0.08398	13	13	241	0.06618	0.07677	12	12	192	0.0559	0.06344
**DATE**															
1987	38	30	77	0.01307	0.01666	0	NA	NA	NA	NA	0	NA	NA	NA	NA
1990	11	10	60	0.01472	0.01864	0	NA	NA	NA	NA	0	NA	NA	NA	NA
1996	22	21	66	0.01284	0.01692	0	NA	NA	NA	NA	0	NA	NA	NA	NA
1997	49	47	183	0.03227	0.0375	0	NA	NA	NA	NA	0	NA	NA	NA	NA
1998	19	16	114	0.01698	0.02754	0	NA	NA	NA	NA	0	NA	NA	NA	NA
1999	117	109	306	0.03949	0.0393	0	NA	NA	NA	NA	0	NA	NA	NA	NA
2000	350	263	479	0.03788	0.05151	1	NA	NA	NA	NA	0	NA	NA	NA	NA
2001	220	173	400	0.03225	0.04716	0	NA	NA	NA	NA	0	NA	NA	NA	NA
2002	254	192	483	0.04924	0.06361	0	NA	NA	NA	NA	0	NA	NA	NA	NA
2003	158	130	328	0.04532	0.0538	2	2	48	0.05263	0.05263	0	NA	NA	NA	NA
2004	183	126	354	0.04267	0.05251	0	NA	NA	NA	NA	1	NA	NA	NA	NA
2005	8	8	172	0.06214	0.07291	0	NA	NA	NA	NA	1	NA	NA	NA	NA
2006	6	6	145	0.0659	0.06982	1	NA	NA	NA	NA	0	NA	NA	NA	NA
2007	15	15	191	0.04953	0.06561	4	4	74	0.04702	0.04529	0	NA	NA	NA	NA
2008	15	15	236	0.05976	0.07967	2	2	49	0.05438	0.05438	1	NA	NA	NA	NA
2009	23	23	277	0.05836	0.0776	2	2	67	0.07306	0.07306	2	2	41	0.06376	0.06386
2010	23	23	281	0.05671	0.07723	8	8	180	0.06538	0.0712	2	2	31	0.04641	0.04641
2011	580	65	607	0.05528	0.08359	48	48	408	0.06444	0.08874	18	18	223	0.05199	0.06321
2012	429	77	588	0.05623	0.08437	41	40	401	0.0674	0.09022	14	14	223	0.05729	0.06786
2013	292	59	543	0.05605	0.0827	29	29	340	0.06419	0.08257	6	6	134	0.06069	0.05992
2014	152	126	501	0.05882	0.08671	10	10	223	0.06691	0.07791	2	2	66	0.06483	0.06483
2015	121	112	465	0.05682	0.08485	8	8	197	0.06855	0.07456	1	NA	NA	NA	NA

*N, number of sequences; h, number of haplotypes; S, number of polymorphic sites; π, nucleotide diversity; θ (W), Watterson's genetic diversity*.

**Figure 2:**
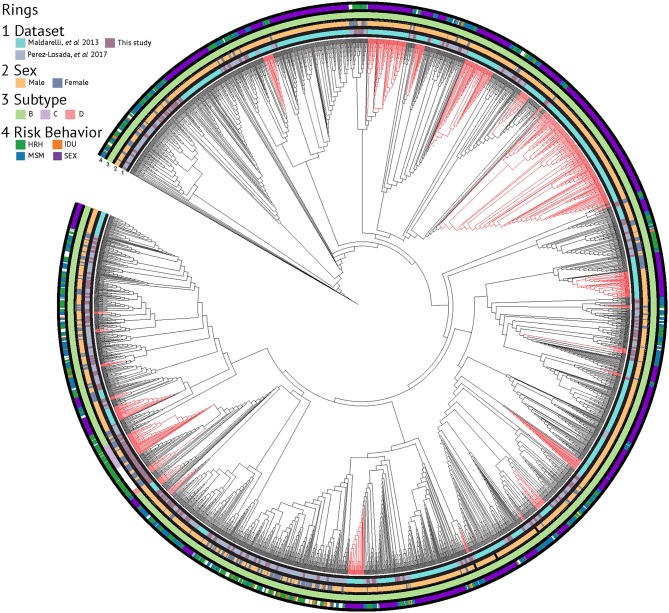
RAxML phylogenetic tree annotated with demographic characteristics. Clusters supported by ≥70% bootstrap values and including ≥5 sequence members are highlighted in red.

**Figure 3:**
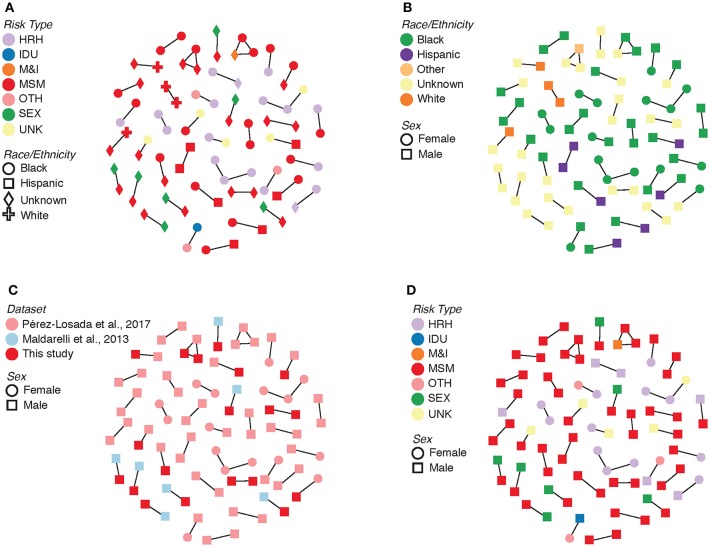
Transmission clusters with associated demographic information. **(A)** Risk type by race/ethnicity. **(B)** Race/ethnicity by sex. **(C)** Dataset by sex. **(D)** Risk type by sex. HRH, high risk heterosexual; IDU, injection drug user; MSM, man who has sex with men; SEX, sexual activity (general); MSM&IDU, MSM and IDU; OTH, other; UNK, unknown.

Evolutionary relationships amongst the HIV sequences were represented by a star phylogeny, indicating equal dispersal around the DC area (Figure 2). We annotated the phylogenetic tree with four demographic characteristics (dataset, sex, subtype, and risk behavior) and found near even dispersal for all of them. Sequences from each of the 33 Maldarelli et al.'s patients clustered together both in RAxML with high support and in HIV-TRACE, as expected; however, some of the intra-patient sequences clustered into more than one cluster. Clusters that were comprised of sequences from a single Maldarelli et al.'s patient were not included in the total cluster number count (RAxML = 27, HIV-TRACE = 30). A total of 1,798 sequences (53.7%) were grouped into 215 clusters in the RAxML tree, with 16 of them belonging to groups with >5 individuals (Figure 2). Of the clusters that were comprised of Maldarelli et al.'s patients, 12 clusters contained at least one sequence from a different dataset (most often the new data). MSM comprised 59.7 and 85.7% of all clusters and clusters with ≥5 individuals, respectively (Table 3). A total of 40 clusters were comprised of MSM and HRH samples, while only 8 contained an IDU sample (Table 3). HIV-TRACE transmission networks were labeled by combinations of three phenotypic characteristics: race, risk factor, and sex (Figure 3, Table 3). A total of 314 (9.4%) sequences were grouped into 41 clusters comprised of two or three individuals. Of these clusters, 78.0% included at least one MSM individual and 31.7% included at least one HRH individual. A third (27.9–31.7%) of the clusters predicted from either method included ≥2 individuals from different datasets. The longest spread of time for a single cluster found in HIV-TRACE and RAxML was 5 years (2007–2012) and 27 years (1987–2014), respectively. The majority of sequences in clusters found for HIV-TRACE methods were from the same year (61%), unlike RAxML, where only a quarter (27.4%) of the sequences in the transmission clusters were from the same year. Overall, RAxML included more individuals in more transmission clusters compared to those predicted by HIV-TRACE.

**Table 3:** Phenotypic characteristics in transmission clusters.

**Trait**	**HIV-TRACE clusters containing ≥1 individual (% of total)**	**RAxML phylogenetic clusters containing ≥1 individual (% of total)**
**Total clusters**	**41**	**215**
**SEX**		
2 individuals	37 (90.2)	144 (67.0)
3 individuals	4 (9.8)	44 (20.5)
4 individuals	0 (0.0)	11 (5.1)
5+ individuals	0 (0.0)	16 (7.4)
**RISK GROUP**		
HRH	13 (31.7)	120 (55.8)
IDU	1 (2.4)	8 (3.7)
M&I	1 (2.4)	7 (3.3)
MSM	32 (78.0)	129 (60.0)
SEX	7 (17.1)	15 (7.0)
Other	3 (7.3)	14 (6.5)
Unknown	5 (12.2)	67 (31.2)
**SEX**		
Female	10 (24.4)	108 (50.2)
Male	37 (78.7)	179 (83.3)
**DATASET**		
(Maldarelli et al., 2013)	7 (17.1)	15 (7.0)
(Pérez-Losada et al., 2017)	32 (78.0)	195 (90.7)
This study	16 (39.0)	68 (31.6)
**RACE**		
Black	27 (65.9)	177 (82.3)
Hispanic	6 (14.6)	18 (8.4)
Other	1 (2.4)	8 (3.7)
Unknown	12 (29.3)	73 (34.0)
White	3 (7.3)	26 (12.1)

*HRH, high risk heterosexual; IDU, injection drug user; MSM, men who have sex with men; M&I, men who have sex with men and injection drug user; SEX, sexual activity (general)*.

**Figure 4:**
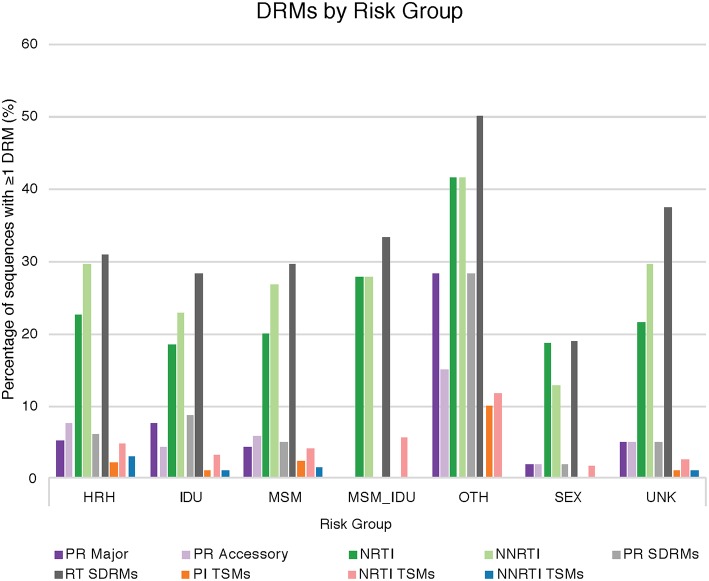
Drug resistance mutations by risk group. The percentage of sequences with at least one drug resistance mutation in a given ART drug category provided by Stanford HIVdb, separated by HIV risk group. HRH, high risk heterosexual; IDU, injection drug user; MSM, man who has sex with men; SEX, sexual activity (general); MSM_IDU, MSM and IDU; OTH, other; UNK, unknown; PR Major, DRMs that make a major contribution to reduced susceptibility to protease inhibitors; PR Accessory, DRMs that contribute to reduced susceptibility in combination with PR Major DRMs; NRTI, nucleoside reverse transcriptase inhibitors; NNRTI, non-nucleoside reverse transcriptase inhibitors; PR SDRMs, protease surveillance drug resistant mutations; RT SDRMs, reverse transcriptase surveillance drug resistant mutations; PI TSMs, protease inhibitor treatment-selected mutations; NRTI TSMs, nucleoside reverse transcriptase inhibitors treatment-selected mutations; NNRTI TSMs, non-nucleoside reverse transcriptase inhibitors treatment-selected mutations.

**Figure 5:**
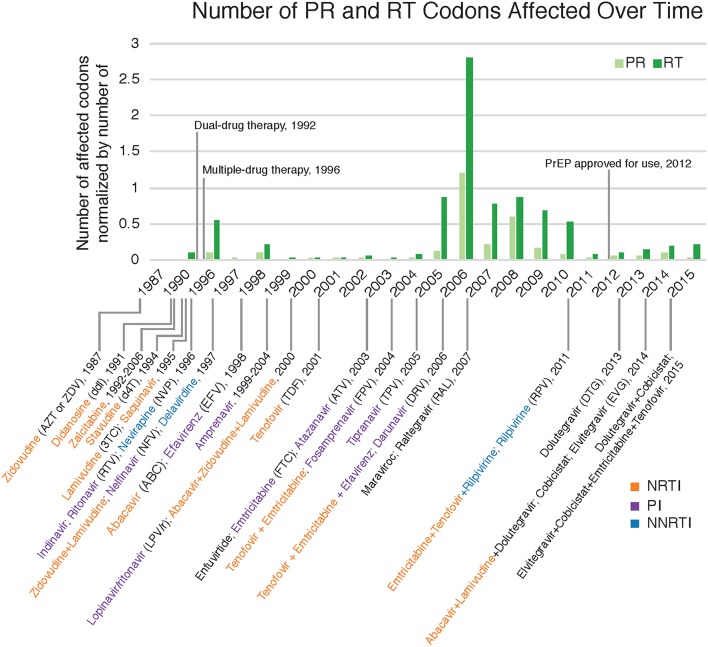
Drug resistant mutations annotated with relevant ART dates. Count of DRM-affected codons observed in each year normalized by the total number of sequences collected in that year. Dates with relevant drug therapy and introductions were included (Arts et al., 2012; Barré-Sinoussi et al., 2013; U.S. Food Drug Administration, 2018). NRTI, nucleoside reverse transcriptase inhibitors; PI, protease inhibitors; NNRTI, non-nucleoside reverse transcriptase inhibitors.

Drug resistance mutations (DRMs) were analyzed for subtype B sequences only (95% of the total sequence data). Across the combined dataset comprising all three studies, 24 amino acids in *PR* and 61 in *RT* were affected by drug resistance mutations. The types of antiretroviral drugs with the highest DRM prevalence were nucleoside reverse transcriptase inhibitors (NRTI), non-nucleoside reverse transcriptase inhibitors (NNRTI), and reverse transcriptase surveillance drug resistance mutations (RT SDRMs); these drugs were found to have between 64 and 97 unique DRMs present in our dataset (Figure 4, Table 4), with unique DRMs referring to a specific DRM observed at a codon position in one or more sequences. Therefore, if a mutation was detected more than once it was not double counted. Additionally, more than 20% of the individuals in the combined dataset displayed at least one DRM for one or more of these antiretroviral (ART) drugs. The risk group with the highest prevalence of DRMs was blood transfusion/perinatal (58.33%), but this is likely due to the low number of blood transfusion/perinatal individuals in our dataset (44 individuals, 1.3%). Additionally, these individuals often begin ART regimens almost immediately upon birth or acquisition of HIV, thus providing a longer time for DRMs to develop and persist in their viral population. Heterosexuals and MSM were found to have similar DRM prevalence at 43.94 and 40.13%, respectively. Those individuals with unknown risk factors had a DRM prevalence of 44.26%, and a lower prevalence was seen in injection drug users at 33.33% (Figure 4). Notably, a 60% increase in the number of codons affected by DRMs occurred between 2006 and 2010 (Figure 5). The amount of DRMs for PR Major (DRMs that make a major contribution to reduced susceptibility to protease inhibitors), PR Accessory (DRMs that contribute to reduced susceptibility in combination with PR Major DRMs), PR SDRMs, NRTI, NNRTI, RT SDRMs, and protease inhibitor treatment-selected mutations (PI TSMs) increased notably in the more recent years (2011–2015); however, this is likely due to the high number of sequences included in those years (Table 4). NRTI and RT SDRMs consistently had higher number of sequences that contained ≥1 DRM, total mutations, and unique mutations from 1996 onward.

**Table 4:** Drug resistant mutations over time.

		**1987**			**1990**			**1996**			**1997**		
**Number of sequences (total)**		**38**			**11**			**22**			**49**		
		**N**	**TM**	**UM**	**N**	**TM**	**UM**	**N**	**TM**	**UM**	**N**	**TM**	**UM**
Protease	PR Major	0	0	0	0	0	0	22	22	1	0	0	0
	PR Accessory	0	0	0	0	0	0	22	22	1	1	1	1
	PR SDRMs	0	0	0	0	0	0	22	44	2	0	0	0
Reverse Transcriptase	NRTI	0	0	0	0	0	0	22	86	7	0	0	0
	NNRTI	0	0	0	1	1	1	22	40	5	0	0	0
	RT SDRMs	0	0	0	1	1	1	22	111	10	0	0	0
	PI TSMs	0	0	0	0	0	0	1	1	1	0	0	0
	NRTI TSMs	0	0	0	0	0	0	1	1	1	0	0	0
	NNRTI TSMs	0	0	0	0	0	0	0	0	0	0	0	0
		**1998**			**1999**			**2000**			**2001**		
**Number of sequences (total)**		**19**			**117**			**350**			**220**		
Protease	PR Major	1	1	1	0	0	0	3	3	3	3	3	3
	PR Accessory	1	1	1	0	0	0	1	1	1	0	0	0
	PR SDRMs	1	1	1	0	0	0	3	3	3	3	3	3
Reverse Transcriptase	NRTI	17	18	3	19	20	4	139	139	1	13	13	3
	NNRTI	1	1	1	21	21	1	4	4	3	2	2	2
	RT SDRMs	17	19	4	17	17	1	142	142	3	13	13	3
	PI TSMs	0	0	0	0	0	0	0	0	0	0	0	0
	NRTI TSMs	0	0	0	0	0	0	1	1	1	0	0	0
	NNRTI TSMs	0	0	0	0	0	0	1	1	1	1	1	1
		**2002**			**2003**			**2004**			**2005**		
**Number of sequences (total)**		**254**			**158**			**183**			**8**		
Protease	PR Major	0	0	0	0	0	0	1	1	1	0	0	0
	PR Accessory	4	4	4	0	0	0	2	2	2	2	2	2
	PR SDRMs	0	0	0	0	0	0	1	1	1	0	0	0
Reverse Transcriptase	NRTI	29	31	7	2	3	2	24	24	2	4	6	5
	NNRTI	7	7	6	54	54	4	76	80	9	1	1	1
	RT SDRMs	30	33	8	5	6	3	24	28	6	3	5	4
	PI TSMs	0	0	0	0	0	0	2	2	2	0	0	0
	NRTI TSMs	1	1	1	1	1	1	20	20	1	1	1	1
	NNRTI TSMs	1	1	1	0	0	0	0	0	0	0	0	0
		**2006**			**2007**			**2008**			**2009**		
**Number of sequences (total)**		**5**			**14**			**15**			**23**		
Protease	PR Major	1	4	4	0	0	0	1	4	4	1	1	1
	PR Accessory	2	2	2	3	3	3	4	6	6	3	3	3
	PR SDRMs	2	5	5	0	0	0	3	7	6	1	2	2
Reverse Transcriptase	NRTI	2	11	9	3	6	6	5	9	7	5	10	8
	NNRTI	2	2	2	4	5	3	6	9	7	7	10	6
	RT SDRMs	3	12	10	5	10	8	6	12	8	7	16	10
	PI TSMs	0	0	0	0	0	0	0	0	0	0	0	0
	NRTI TSMs	1	2	2	1	1	1	0	0	0	3	3	3
	NNRTI TSMs	1	1	1	1	1	1	0	0	0	2	2	2
		**2010**			**2011**			**2012**			**2013**		
**Number of sequences (total)**		**23**			**585**			**431**			**295**		
Protease	PR Major	0	0	0	33	63	22	25	43	20	16	37	12
	PR Accessory	3	3	2	42	61	20	24	35	16	14	19	11
	PR SDRMs	0	0	0	34	79	23	31	57	20	17	37	13
Reverse Transcriptase	NRTI	9	10	7	131	226	64	89	171	57	65	131	44
	NNRTI	11	15	9	166	258	55	126	191	51	85	138	40
	RT SDRMs	11	17	10	191	377	51	140	276	48	90	212	42
	PI TSMs	0	0	0	16	16	7	10	10	6	8	9	4
	NRTI TSMs	0	0	0	25	27	9	16	16	6	17	21	7
	NNRTI TSMs	2	2	1	6	7	6	7	8	5	4	4	2
		**2014**			**2015**								
**Number of sequences (total)**		**151**			**121**								
Protease	PR Major	10	20	16	5	6	3						
	PR Accessory	7	10	9	1	1	1						
	PR SDRMs	10	22	13	5	7	4						
Reverse Transcriptase	NRTI	26	49	29	21	32	16						
	NNRTI	35	51	23	30	38	15						
	RT SDRMs	46	81	30	31	50	17						
	PI TSMs	3	3	2	0	0	0						
	NRTI TSMs	4	5	3	2	3	3						
	NNRTI TSMs	0	0	0	4	4	4						

*N, number of sequences with ≥1 DRM; TM, total number of mutations; UM, number of unique mutations; PR Major, DRMs that make a major contribution to reduced susceptibility to protease inhibitors; PR Accessory, DRMs that contribute to reduced susceptibility in combination with PR Major DRMs; PR SDRMs, protease surveillance drug resistant mutations; NRTI, nucleoside reverse transcriptase inhibitors; NNRTI, non-nucleoside reverse transcriptase inhibitors; RT SDRMs, reverse transcriptase surveillance drug resistant mutations; PI TSMs, protease inhibitor treatment-selected mutations; NRTI TSMs, nucleoside reverse transcriptase inhibitors treatment-selected mutations; NNRTI TSMs, non-nucleoside reverse transcriptase inhibitors treatment-selected mutations*.

**Figure 6:**
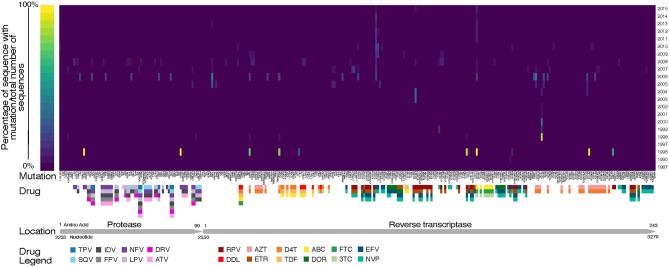
Heat map of drug resistant mutations over time. Annotated with genome location, relative to HXB2 reference, and known drugs that the mutations are selected by. TPV, Tipranavir; SQV, Saquinavir; NFV, Nelfinavir; LPV, Lopinavir; IDV, Indinavir; DRV, Darunavir; ATV, Atazanavir; RPV, Rilpivirine; DDL, Didanosine; AZT, Zidovudine; ETR, Etravirine; D4T, Stavudine; TDF, Tenofovir Disoproxil Fumarate; ABC, Abacavir; DOR, Doravirine; FTC, Emtricitabine; 3TC, Lamivudine; EFV, Efavirenz; NVP, Nevirapine.

Fewer unique mutations were found to be at higher frequencies in the 1990s, whereas more unique mutations were found to be at a lower frequency in the 2000s (Table 4, Figure 6). This trend can be explained by the number of sequences per year, where there were lower total sequences in the 1990s and early 2000s (Table 4). However, despite there being a lower number of total sequences between 2005 and 2010, there were more distinct mutations in that time range (Figure 6) as well as a peak in the number of affected codons (Figure 5), as seen in a recent study of treatment-naïve individuals in the US (Ross et al., 2018). Notably, in 1996 there were eight DRMs found at high frequencies (*PR*: D30N, N88D; *RT*: D67N, K70R, Y181C, M184V, K219E, H22IY); all eight DRMs belonged to a single patient from Maldarelli et al.'s dataset (patient 27), in which one blood sample from a single appointment was obtained and 22 serial dilutions resulted in 22 separate sequences (Maldarelli et al., 2013). Two mutations in *RT*, K103N and M184V, persisted from around 2005 to 2015 at a lower frequency (average 12.4 and 8.8%, respectively). K103N is a Major NNRTI resistant mutation, whereas M184V is a Major NRTI resistant mutation.

## Discussion

### Transmission Networks

Multiple studies have identified transmission networks as playing a significant role in the spread of HIV in the United States and DC (Magnus et al., 2009; Pérez-Losada et al., 2010, 2017; Esbjörnsson et al., 2016; Kassaye et al., 2016; Morgan et al., 2017; Wertheim et al., 2017). We identified in our dataset 41 and 76 unique transmission clusters using HIV-TRACE and a ML phylogenetic method (RAxML), respectively, most of which included members of the MSM and HRH risk groups. Transmission within these high-risk groups is expected given that both groups are reported as continuing sources of HIV infection in Washington, DC (District of Columbia Department of Health, HIV/AIDS, Hepatitis, STD and TB Administration (HAHSTA), 2017, 2018). A majority of clusters included at least one non-Hispanic black individual, which is consistent with public health surveillance data as well (District of Columbia Department of Health, HIV/AIDS, Hepatitis, STD and TB Administration (HAHSTA), 2018). Like Kassaye et al. (2016), the majority of our identified transmission clusters were comprised of only two or three patients (81.9–100%). The ML and HIV-TRACE results included several transmission clusters containing individuals from different datasets (27.9 and 31.7%, respectively), suggesting that many of those networks are unique to this study. By combining sequencing and demographic information across a 28-year time window, we found that individuals in transmission networks predicted by HIV-TRACE had sequences obtained from different years ranging between 1 and 5 years apart, with a mean of 2.5 year spread in sequence time. However, the majority of the clusters for HIV-TRACE contained sequences from the same year (61.0%). For the RAxML, on the other hand, the longest amount of time between sequences in a transmission cluster was 27 years; although there was a mean of 2.4 year spread in sequence time. These results indicate that viral sequences do not often persist over many years, but when they do, distance-based cluster analyses might have a more difficult time identifying such transmission networks compared to evolutionary phylogenetic approaches. Our results show how insights from longitudinal studies can be used to understand viral dynamics over time in a local epidemic as well as how methodological choices can impact final inferences in such studies.

### Temporal Diversity of HIV in DC

HIV diversity, as indicated by the number of unique subtypes and recombinants, and genetic diversity increased in the latter years (most recent sampling) of our study. Subtype B (94.95%) and B recombinants (2.9%) dominated our HIV dataset, followed by subtype C (1.43%). As suggested by Grossman et al. (2018), subtype C has been in DC since the late 1980s; however, our sampling of those same years did not include subtype C sequences. Similarly to a recent study of the San Francisco area over a 14-year time period, we also found over 4% non-subtype B HIV-1 variants in our combined dataset (Dalai et al., 2018). Focusing on subtype B, genetic diversity was similar across race/ethnicity but was higher in HRH and MSM relative to IDU risk factors. Genetic diversity increased steadily over time with a slight peak in 2009 and remained constant thereafter until 2015 (θ = ~0.086). Additionally, π, which is indicative of current genetic diversity (Crandall et al., 1999), increased over time as well, further suggesting that variants are accumulating in the DC population. The number of haplotypes generally increased until 2005 followed by a sharp reduction of haplotypes but a consistent genetic diversity. This trend could be a consequence of the small sample sizes in years 2005–2010, but relatively low numbers of haplotypes remained in years 2011–2013 despite high sample sizes. Our phylodynamic analyses of the three dominant subtypes in our dataset (B, B recombinants, and C) showed that the relative genetic diversity of DC's HIV population has remained relatively steady over the last 35 years, with subtype C showing an increase in relative genetic diversity and subtype B showing an increase in diversity since 2000. These results coupled with the phylogenetic tree being star-like further bolsters the conclusion that the HIV epidemic in DC is mature and individuals with varying infection durations and risk factor have been intermingling for years (Kassaye et al., 2016).

### Evolution of Drug Resistant Mutations in DC

Our study found a higher percentage of patients with DRMs (37.9%) than a previous study (Kassaye et al., 2016) of the DC epidemic (22.5%). This is expected since our study also included a higher proportion of treatment-experienced individuals, while Kassaye et al. involved only ART-naïve patients. The DRM classes designated by the Stanford University HIV Drug Resistance Database (NRTI, NNRTI, and RT SDRMs) were notably more prominent in our data. There was little variation between the number of DRM-affected codons between risk groups.

We found an increase in the number of codons affected by DRMs between 2005 and 2008. It is possible that the smaller number of sequences from the years 2005 through 2010 relative to other years may have contributed to an artificial inflation of the number of DRMs during that time period. Additionally, all sequences from 2005 through 2010 came from the new dataset and were all treatment-naïve. A similar trend and spike in DRM prevalence was found in treatment-naïve patients in the U.S. between 2000 and 2009 (Ross et al., 2018), which correlates with the increase in diversity in subtype B. Such a trend may have been caused by changes in ART recommendations that allowed treatment to begin more quickly after diagnosis, the approval of additional therapies, mainly integrase inhibitors and specifically Maraviroc (MVC) and Raltegravir (RAL) introduced in 2007 (see Figure 5), and the increased use of genotypic screening for drug resistance prior to treatment (Ross et al., 2018). Kassaye et al. (2016) also observed a downward trend in DRMs over time between 1997–2006 and 2007–2013. Furthermore, medication guidelines recommended by physicians to patients have changed over time, which could result in the variation of number of DRMs and number of codon positions affected by mutation. Moreover, for the treatment-experienced individuals, HIV sequencing was likely requested because of concerns about their adherence and presumably because they are still viremic while being on medication, contributing to the increase in these numbers as well.

Although there was a decline in the number of DRM-affected codons around 2008, the number of unique drug resistant mutations increased throughout the 2000s. All of these DRMs were found at low frequencies, but a similar upward trend was also seen in genetic and nucleotide diversity. With increased diversity and increased DRMs, combating HIV replication and spread becomes more cumbersome, thus requiring researchers and doctors to stay one step ahead of the epidemic by developing new drugs that are not already impaired by the current DRMs before the current drugs are no longer effective. This is partially corrected already by patients in dual- or multiple-class ART regimens. Two mutations, K103N and M184V both in *RT*, persisted for 10 years in this combined dataset. K103N is selected for by usage of nevirapine (NVP) and efavirenz (EFV) (Bacheler et al., 2000; Gulick et al., 2004; Margot et al., 2006); M184V is selected by lamivudine (3TC) and emtricitabine (FTC) (Keulen et al., 1996; Frost et al., 2000). These four drugs are commonly prescribed as part of treatment recommendations (Geneva: World Health Organization, 2016, 2017).

### Limitations

This study has a few limitations. Sampling across years is inconsistent and heterogeneous with more recent years containing more individuals and HIV sequences than older years. Additionally, initial years of the DC HIV epidemic (1980s) are missing. This uneven sampling could shorten the duration of the observed transmission clusters. This is further compounded when using HIV-TRACE, because a genetic distance cut-off is used, limiting the transmission clusters predicted by HIV-TRACE to more similar sequences. We supplemented the transmission clusters predicted by HIV-TRACE with transmission clusters predicted by phylogenetic methods, where genetic distance is not a cut-off (only branch support is), thus allowing for more distantly related viruses to be included in predicted transmission clusters. Each dataset was sequenced in different laboratories, leading to potential biases in ambiguity codes and consensus sequence resolution that could affect diversity estimates and potentially produce false positive or negative transmission clusters. Moreover, the direction of HIV virus transmission in a transmission cluster was not determined.

## Conclusions

Like several other studies in the US (Latkin et al., 2011; DiNenno et al., 2012; Raymond et al., 2017) and in DC (Kassaye et al., 2016), our study supports the DC DOH's observation that HRH and MSM risk groups are primarily contributing to transmission of HIV (District of Columbia Department of Health, HIV/AIDS, Hepatitis, STD and TB Administration (HAHSTA), 2017, 2018). As in our study, the majority of identified transmission clusters include at least one MSM and one HRH individual and often involve sequences obtained from the same year. Additionally, insights from DRM prevalence and genetic diversity trends over time will allow treatment measures to be more effective. Specifically, we found that both genetic diversity and the number of unique DRMs in circulation increased in subtype B sequences from more recent years. As such, our findings suggest that new surveillance studies of HIV subtypes should be conducted to better understand the current transmission dynamics of HIV in Washington, DC, especially in treatment-naïve individuals where our study and others (Kassaye et al., 2016; Ross et al., 2018) saw a spike between 2005 and 2008 in DRM.

## Data Availability

The new HIV sequence data associated with this study have been deposited in GenBank under accession numbers MK270625 - MK270927.

## Ethics Statement

Individuals represented in the new HIV data participated in clinical protocols (00-I-0110, 97-I-0082, and 95-I-0072), which took place at the NIH Clinical Center in Bethesda, Maryland. These protocols were approved by the NIAID Institutional Review Board (FWA00005897). All participants provided written informed consent.

## Author Contributions

KC, MP-L, ZG, FM, and SK designed the project. SK, ZG, and FM collected data. KG and MS analyzed the data and performed all bioinformatic analyses. MP-L and KC reviewed all analyses. KG, MS, MP-L, and KC prepared original draft of manuscript, and all authors read, revised and approved the final manuscript.

### Conflict of Interest Statement

The authors declare that the research was conducted in the absence of any commercial or financial relationships that could be construed as a potential conflict of interest.
