# Corrigendum: A 28-Year History of HIV-1 Drug Resistance and Transmission in Washington, DC

**DOI:** 10.3389/fmicb.2019.02590

**Published:** 2019-11-12

**Authors:** Keylie M. Gibson, Margaret C. Steiner, Seble Kassaye, Frank Maldarelli, Zehava Grossman, Marcos Pérez-Losada, Keith A. Crandall

**Affiliations:** ^1^Computational Biology Institute, Milken Institute School of Public Health, George Washington University, Washington, DC, United States; ^2^Department of Medicine, Georgetown University, Washington, DC, United States; ^3^HIV Dynamics and Replication Program, Host-Virus Interaction Branch, Center for Cancer Research, National Cancer Institute, National Institutes of Health, Bethesda, MD, United States; ^4^Sackler Faculty of Medicine, School of Public Health, Tel Aviv University, Tel Aviv, Israel; ^5^CIBIO-InBIO, Centro de Investigação em Biodiversidade e Recursos Genéticos, Universidade do Porto, Vairão, Portugal; ^6^Department of Epidemiology and Biostatistics, Milken Institute School of Public Health, George Washington University, Washington, DC, United States

**Keywords:** Washington, DC, phylodynamics, HIV-1, drug resistance mutations, transmission networks

In the original article, there was a mistake in Table 4. The first column headings on page 12 of the PDF, labeled “1987, 1990, 1996, and 1997,” should in fact be labeled “2010, 2011, 2012, and 2013,” respectively. The corrected portion of Table 4 appears below.

**Table 4:** 

		**2010**			**2011**			**2012**			**2013**		
**Number of sequences (total)**		**38**			**11**			**22**			**49**		
		**N**	**TM**	**UM**	**N**	**TM**	**UM**	**N**	**TM**	**UM**	**N**	**TM**	**UM**
	PR SDRMs	0	0	0	34	79	23	31	57	20	17	37	13
Reverse Transcriptase	NRTI	9	10	7	131	226	64	89	171	57	65	131	44
	NNRTI	11	15	9	166	258	55	126	191	51	85	138	40
	RT SDRMs	11	17	10	191	377	51	140	276	48	90	212	42
	PI TSMs	0	0	0	16	16	7	10	10	6	8	9	4
	NRTI TSMs	0	0	0	25	27	9	16	16	6	17	21	7
	NNRTI TSMs	2	2	1	6	7	6	7	8	5	4	4	2

Additionally, in the **Abstract**, the number of individuals in the DC area has been corrected from 1996 to 1995. The fully corrected **Abstract** appears below:

Washington, DC consistently has one of the highest annual rates of new HIV-1 diagnoses in the United States over the last 10 years. To guide intervention and prevention strategies to combat DC HIV infection, it is helpful to understand HIV transmission dynamics in a historical context. Toward this aim, we conducted a retrospective study (years 1987–2015) of 3,349 HIV *pol* sequences (1,026 bp) from 1,995 individuals living in the DC area belonging to three different cohorts. We coupled HIV sequence data with clinical information (sex, risk factor, race/ethnicity, viral load, subtype, anti-retroviral regimen) to identify circulating drug resistant mutations (DRM) and transmission clusters and assess their persistence over time. Of the transmission clusters identified in the DC area, 78.0 and 31.7% involved MSM and heterosexuals, respectively. The longest spread of time for a single cluster was 5 years (2007–2012) using a distance-based network inference approach and 27 years (1987–2014) using a maximum likelihood phylogenetic approach. We found eight subtypes and nine recombinants. Genetic diversity increased steadily over time with a slight peak in 2009 and remained constant thereafter until 2015. Nucleotide diversity also increased over time while relative genetic diversity (BEAST) remained relatively steady over the last 28 years with slight increases since 2000 in subtypes B and C. Sequences from individuals on drug therapy contained the highest total number of DRMs (1,104–1,600) and unique DRMs (63–97) and the highest proportion (>20%) of resistant individuals. Heterosexuals (43.94%), MSM (40.13%), and unknown (44.26%) risk factors showed similar prevalence of DRMs, while injection drug users had a lower prevalence (33.33%). Finally, there was a 60% spike in the number of codons with DRMs between 2007 and 2010. Past patterns of HIV transmission and DRM accumulation over time described here will help to predict future efficacy of ART drugs based on DRMs persisting over time and identify risk groups of interest for prevention and intervention efforts within the DC population. Our results show how longitudinal data can help to understand the temporal dynamics of HIV-1 at the local level.

Finally, in the **Results** section the DRM prevalence for MSM was mistakenly stated as 44.26%. The correct value is 40.13%. A correction has been made to the **Results** section, paragraph 4.

Drug resistance mutations (DRMs) were analyzed for subtype B sequences only (95% of the total sequence data). Across the combined dataset comprising all three studies, 24 amino acids in *PR* and 61 in *RT* were affected by drug resistance mutations. The types of antiretroviral drugs with the highest DRM prevalence were nucleoside reverse transcriptase inhibitors (NRTI), non-nucleoside reverse transcriptase inhibitors (NNRTI), and reverse transcriptase surveillance drug resistance mutations (RT SDRMs); these drugs were found to have between 64 and 97 unique DRMs present in our dataset (Figure 4, Table 4), with unique DRMs referring to a specific DRM observed at a codon position in one or more sequences. Therefore, if a mutation was detected more than once it was not double counted. Additionally, more than 20% of the individuals in the combined dataset displayed at least one DRM for one or more of these antiretroviral (ART) drugs. The risk group with the highest prevalence of DRMs was blood transfusion/perinatal (58.33%), but this is likely due to the low number of blood transfusion/perinatal individuals in our dataset (44 individuals, 1.3%). Additionally, these individuals often begin ART regimens almost immediately upon birth or acquisition of HIV, thus providing a longer time for DRMs to develop and persist in their viral population. Heterosexuals and MSM were found to have similar DRM prevalence at 43.94 and 40.13%, respectively. Those individuals with unknown risk factors had a DRM prevalence of 44.26%, and a lower prevalence was seen in injection drug users at 33.33% (Figure 4). Notably, a 60% increase in the number of codons affected by DRMs occurred between 2006 and 2010 (Figure 5). The amount of DRMs for PR Major (DRMs that make a major contribution to reduced susceptibility to protease inhibitors), PR Accessory (DRMs that contribute to reduced susceptibility in combination with PR Major DRMs), PR SDRMs, NRTI, NNRTI, RT SDRMs, and protease inhibitor treatment-selected mutations (PI TSMs) increased notably in the more recent years (2011–2015); however, this is likely due to the high number of sequences included in those years (Table 4). NRTI and RT SDRMs consistently had higher number of sequences that contained ≥1 DRM, total mutations, and unique mutations from 1996 onward.

The authors apologize for these errors and state that this does not change the scientific conclusions of the article in any way. The original article has been updated.

